# Cannabis use in pregnancy and maternal and infant outcomes: A Canadian cross-jurisdictional population-based cohort study

**DOI:** 10.1371/journal.pone.0276824

**Published:** 2022-11-23

**Authors:** Sabrina Luke, Amy J. Hobbs, Michaela Smith, Catherine Riddell, Phil Murphy, Calypse Agborsangaya, Christina Cantin, John Fahey, Kenny Der, Ann Pederson, Chantal Nelson, on behalf of the National Maternal Cannabis Working Group

**Affiliations:** 1 Perinatal Services British Columbia, Provincial Health Services Authority, Vancouver, British Columbia, Canada; 2 Women’s Health Research Institute, BC Women’s Hospital + Health Centre, Provincial Health Services Authority, Vancouver, British Columbia, Canada; 3 Johns Hopkins University, Baltimore, Maryland, United States of America; 4 Better Outcomes Registry Network Ontario, Ottawa, Ontario, Canada; 5 Children’s & Women’s Health Program, Eastern Health, St. John’s, Newfoundland and Labrador, Canada; 6 Alberta Health, Edmonton, Alberta, Canada; 7 Champlain Maternal Newborn Regional Program, Ottawa, Ontario, Canada; 8 Reproductive Care Program of Nova Scotia, Halifax, Nova Scotia, Canada; 9 Public Health Agency of Canada, Ottawa, Ontario, Canada; University of Tennessee Knoxville, UNITED STATES

## Abstract

**Background:**

With the recent legalization of cannabis in Canada, there is an urgent need to understand the effect of cannabis use in pregnancy. Our population-based study investigated the effects of prenatal cannabis use on maternal and newborn outcomes, and modification by infant sex.

**Methods:**

The cohort included 1,280,447 singleton births from the British Columbia Perinatal Data Registry, the Better Outcomes Registry & Network Ontario, and the Perinatal Program Newfoundland Labrador from April 1st, 2012 to March 31st, 2019. Logistic regression determined the associations between prenatal cannabis use and low birth weight, small-for-gestational age, large-for-gestational age, spontaneous and medically indicated preterm birth, very preterm birth, stillbirth, major congenital anomalies, caesarean section, gestational diabetes and gestational hypertension. Models were adjusted for other substance use, socio-demographic and-economic characteristics, co-morbidities. Interaction terms were included to investigate modification by infant sex.

**Results:**

The prevalence of cannabis use in our cohort was approximately 2%. Prenatal cannabis use is associated with increased risks of spontaneous and medically indicated preterm birth (1.80[1.68–1.93] and 1.94[1.77–2.12], respectively), very preterm birth (1.73[1.48–2.02]), low birth weight (1.90[1.79–2.03]), small-for-gestational age (1.21[1.16–1.27]) and large-for-gestational age (1.06[1.01–1.12]), any major congenital anomaly (1.71[1.49–1.97]), caesarean section (1.13[1.09–1.17]), and gestational diabetes (1.32[1.23–1.42]). No association was found for stillbirth or gestational hypertension. Only small-for-gestational age (p = 0.03) and spontaneous preterm birth (p = 0.04) showed evidence of modification by infant sex.

**Conclusions:**

Prenatal cannabis use increases the likelihood of preterm birth, low birth weight, small-for-gestational age and major congenital anomalies with prenatally exposed female infants showing evidence of increased susceptibility. Additional measures are needed to inform the public and providers of the inherent risks of cannabis exposure in pregnancy.

## Introduction

The Government of Canada passed the Cannabis Act (Bill C-45) on October 17^th^, 2018, legalizing the possession, distribution, sale and production of cannabis in Canada [1]. Approximately 15% of Canadian women of childbearing age reported cannabis use in the year prior to legalization, a trend that is expected to increase with the changing legislation [2–4]. Cannabinoids readily cross the human placenta, potentially causing both immediate and delayed health effects [5]. It is hypothesized that prenatal exposure to cannabis produces similar outcomes to tobacco by reducing blood flow to the placenta [6]. Limited research has reported that prenatal cannabis exposure adversely impacts neurodevelopment in male fetuses but not female fetuses through growth restriction [7–13]. Evidence suggests potential sex differences in the affinity of cannabinoid receptor 1 in the brain, the main target of Δ9-Tetrahydrocannabinol [12].

Given the changes in legislation, trends in use, and limited safety information in pregnancy, there is an urgent need to determine the potential impact of prenatal cannabis exposure to fetal development and maternal health.

While Canada has long engaged in routine surveillance of alcohol and tobacco use, there is currently no ongoing, standardized national surveillance system that collects, analyzes, and disseminates information on cannabis use during pregnancy. The Public Health Agency of Canada, the Canadian Perinatal Programs Coalition, and Perinatal Services BC led a collaboration of perinatal programs across Canada to improve and standardize national data collection of prenatal cannabis use and study the impact of exposure on maternal and infant health.

This study is the first to pool national population-based data from across Canada to investigate the effects of prenatal cannabis use on both maternal and newborn outcomes, and examine whether infant outcomes differ by infant sex.

## Methods

### Participants

Canadian perinatal programs from twelve provinces and territories identified jurisdictional data availability of prenatal cannabis use to investigate its effects on maternal and infant outcomes. Three provinces, British Columbia (BC), Ontario (ON) and Newfoundland and Labrador (NL), were able to contribute both prenatal cannabis use and perinatal data through third-party perinatal data registries including British Columbia’s Perinatal Data Registry, the Better Outcomes Registry & Network Ontario, and Perinatal Program Newfoundland and Labrador for the study period of April 1^st^, 2012 to March 31^st^, 2019. Other provinces and territories did not contribute data for the following reasons: limited funding or capacity to collect data, years of data did not overlap with other jurisdictions, cannabis data could not be linked with perinatal data, and changes in government during the study period prevented data sharing.

### Sources of data

Prenatal cannabis use was collected through maternal self-report documented by providers at antenatal visits and hospital admission for labour and birth. Information on the frequency, dose or route of administration was not available for any jurisdiction. BC only records a positive response to cannabis use on the antenatal record resulting in a dichotomous ‘Yes’ or ‘Unknown’ variable while ON and NL collect “Yes”, “No” and “Unknown” responses. ON asks the question “Have you ever used drugs and/or other substances during your pregnancy?” on their maternal health history questionnaire and includes a multi-select pick list of selected substances to record responses with cannabis use captured as “Marijuana”. BC includes a substance use section in its antenatal form but does not specify a question and also includes a multi-select pick list of substances, with cannabis listed as “Marijuana”. NL includes a section on substance use on their prenatal record but does not include a question specific to cannabis use. Information on cannabis use was collected at any time during pregnancy and did not include pre-conception or postpartum use. While it is recommended, providers are not required to ask about substance use and the manner in which the question is asked may vary between providers. Data collection methods remained consistent for all three jurisdictions during the study time period.

Other substance use variables collected at the antenatal visit by all three jurisdictions included tobacco, alcohol, solvents, cocaine, methadone, heroin and opioids. Heroin, opioids, methadone, cocaine and solvents were combined for the purposes of data analysis.

Gestational age was defined as gestational age at delivery in completed weeks of pregnancy measured by ultrasound at the first prenatal visit, by last menstrual period where ultrasound was not available, or by clinical exam of the infant. BC used an algorithm to identify the most robust measure of gestational age available based on a hierarchy of criteria [14].

Diabetes variables were defined using ICD-10-CA codes and antenatal record risk flags for BC and NL while hypertension variables were defined using only ICD-10-CA codes. S1 Table includes the complete list of ICD-10-CA codes used for diabetes and hypertension. ON used the antenatal record alone as the source of hypertension and diabetes information. Quintile of Annual Income Per Person Equivalent (QAIPPE) score and rurality of maternal residence were identified using Statistics Canada’s Postal Code Conversion File (PCCF+) based on the 2016 census with rurality defined as residence within a community of less than 10,000 residents. QAIPPE is a neighborhood-level index used as a proxy measure for individual-level socioeconomic status.

### Outcomes

The main outcomes of interest included low birth weight (LBW), small-for-gestational age (SGA), large-for-gestational age (LGA), spontaneous (SPTB) and medically indicated preterm birth (MPTB), very preterm birth (VPTB), stillbirth, major congenital anomalies, caesarean section, gestational diabetes and gestational hypertension. Outcomes were chosen as key indicators of fetal growth and development, pregnancy complications and predictors of long term infant and maternal health. Gestational age at birth and birth weight are biomarkers of fetal neurodevelopmental risk [13], a key concern with prenatal cannabis exposure.

SGA and LGA were calculated using a Canadian reference curve developed by Kramer et al. (2001) [15]. SGA was defined as birth weight for sex and gestational age in weeks at delivery below the 10th percentile, while LGA was defined as birth weight for sex and gestational age in weeks at delivery above the 90th percentile. PTB was defined as an infant born before 37 weeks of gestation, while VPTB was defined as birth before 32 weeks of gestation. SPTB indicated that labour began without induction or the use of caesarean section while MPTB indicated that preterm delivery was initiated through the use of medical intervention. LBW was defined as an infant born at a weight <2500g at birth.

In accordance with the Kramer reference, the analyses examining PTB, SGA, LGA and LBW outcomes was restricted to live births between 22 and 43 weeks of gestation and excluded infants where sex could not be determined. The three jurisdictions all applied the same exclusion criteria for gestational age.

Stillbirth was defined as the complete expulsion or extraction of a product of conception after at least 20 weeks of pregnancy or after attaining a weight of at least 500 grams, in which after expulsion or extraction, there is no breathing, beating of the heart, pulsation of the umbilical cord, or unmistakable movement of the voluntary muscle.

Any major congenital anomalies at birth included neural tube defects, other central nervous system defects, selected sense organ defects, selected congenital heart defects, oro-facial clefts, selected gastrointestinal defects, selected urinary tract defects, selected genital anomalies, limb deficiency defects, diaphragmatic hernia, prune belly sequence, selected abdominal wall defects, and selected chromosomal defects. These were included based on the previous work done by the Canadian Congenital Anomalies Surveillance System [16]. These conditions were grouped together for the purposes of the analysis. ICD-10-CA codes are shown in S1 Table.

### Statistical analysis

**Fig 1:**
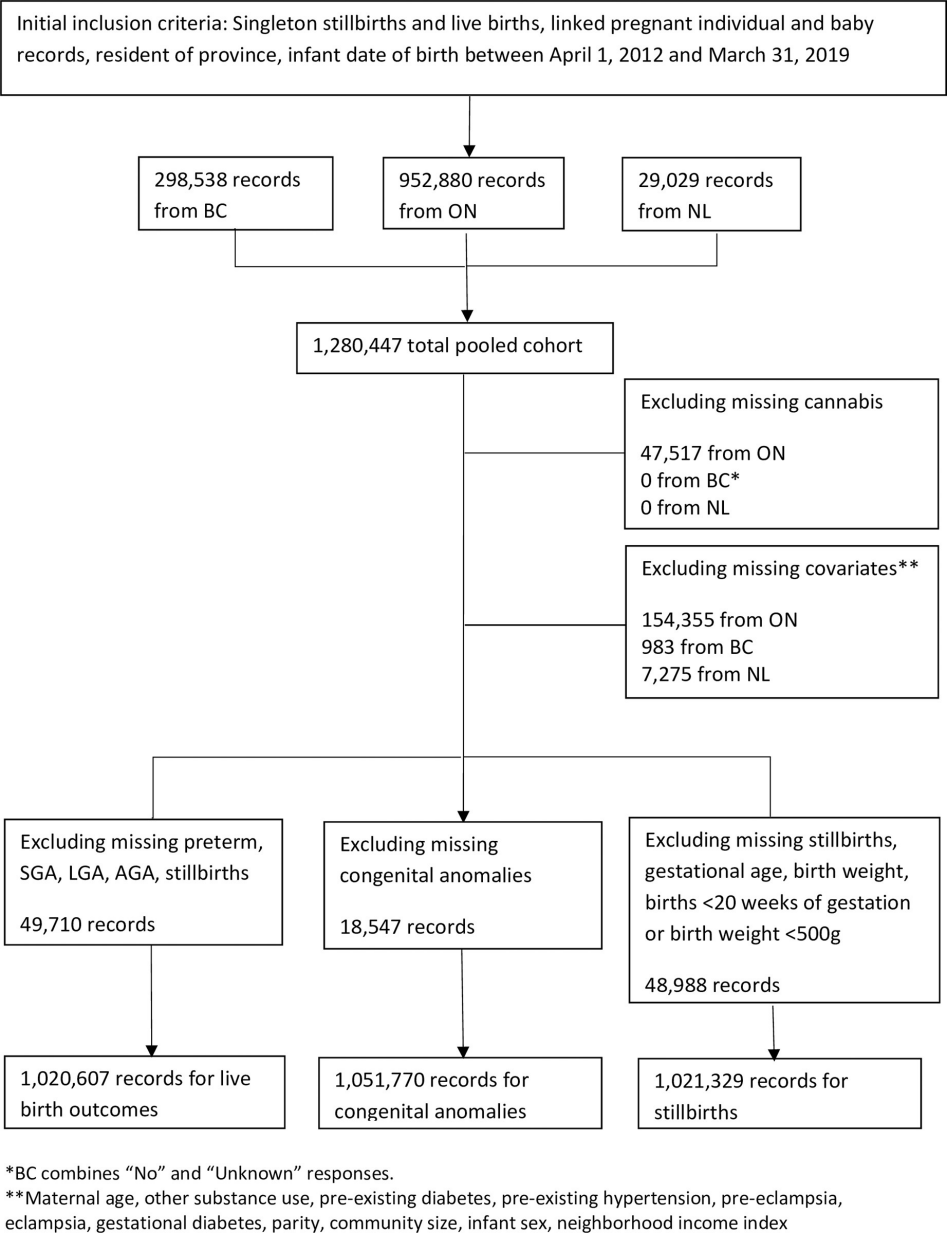
Flowchart of study cohort. * BC combines “No” and “Unknown” responses. ** Maternal age, other substance use, pre-existing hypertension, pre-eclampsia, eclampsia, gestational diabetes, parity, community size, infant sex, neighborhood income index.

Data from the British Columbia Perinatal Data Registry, the Better Outcomes Registry & Network Ontario (BORN), and the Perinatal Program Newfoundland Labrador were transferred to and stored in Population Data British Columbia’s secure research environment for analysis [17]. The initial cohort included only singleton births to pregnant individuals who were residents of the province and where newborn records could be linked. We did not include multiple births in our analysis as newborns are often born smaller and earlier compared to singleton births. Late terminations were excluded from the initial cohort since only BC captures this information within their perinatal data registry. Separate cohorts were created for stillbirth, congenital anomalies and maternal outcomes as shown in Fig 1.

After the initial exclusion criteria, the cohort included 1,280,447 live and stillbirth pregnancies (952,880 from ON, 298,538 from BC, and 29,029 from NL). Records with missing cannabis and covariate data were further excluded, and additional restrictions were applied depending on the outcome of interest as outlined in Fig 1.

A retrospective study was conducted on the final cohort. Chi-square tests were used to compare the maternal characteristics of pregnant individuals who used cannabis with those who did not. The variables were tested for multicollinearity using correlation matrices. Adjusted logistic regressions were performed to determine the associations between cannabis use in pregnancy and maternal and infant outcomes.

Models for infant outcomes were adjusted for other substance use, maternal age, pre-pregnancy BMI, rural maternal residence, parity, QAIPPE score, pre-existing diabetes, chronic hypertension, gestational diabetes and gestational hypertension. Infant sex was added as an interaction term to regression models for newborn outcomes to identify modification by infant sex. Models for maternal outcomes were adjusted for the same factors as for infants above and either gestational diabetes or gestational hypertension depending on the outcome of interest. These were selected based on causal diagrams and a priori subject-matter knowledge. Further sub-analyses were conducted to compare associations in pregnant individuals who only used cannabis with those who used cannabis and other substances. E-values were calculated to estimate the impact of unmeasured confounding [18]. Results are summarized in S2 and S3 Tables.

All analyses were conducted using SAS 9.4. Research ethics board approvals and other data sharing agreements were obtained from participating jurisdictions to access third-party de-identified registry data as well as waive informed consent on the basis of implied consent of registry participants (University of British Columbia Research Ethics Board (H18-03440)).

## Results

We did not find any evidence of multicollinearity among variables. Table 1 compares sociodemographic characteristics and outcomes between pregnant individuals who reported cannabis use and those who did not. The prevalence of cannabis use in our cohort was approximately 2% and increased over time from 1.5% in 2012/2013 to 2.5% in 2018/2019. Pregnant individuals who used cannabis were more frequently age 24 or younger (p<0.0001) and underweight or obese (p<0.0001). There was a high co-occurrence of cannabis use with other substance use. Pregnant individuals who used cannabis more often lived in a rural community and in a neighborhood in the lowest income quintile compared with non-users.

**Table 1:** Maternal characteristics by prenatal cannabis use, British Columbia, Ontario and Newfoundland and Labrador 2012–2019.

Maternal characteristics	Cannabis use		No cannabis use		p-value
	(N = 20,410)		(N = 1,031,360)		
	n	%	n	%	
Fiscal Year^a^					
2012/2013	2,187	1.5	142,313	98.5	<0.0001
2013/2014	2,310	1.6	142,687	98.4	
2014/2015	2,788	1.9	142,017	98.1	
2015/2016	2,782	1.8	150,051	98.2	
2016/2017	3,129	2.1	148,222	97.9	
2017/2018	3,271	2.2	147,695	97.8	
2018/2019	3,607	2.5	143,360	97.6	
Maternal age					
<20	2,329	11.4	15,859	1.5	<0.0001
20–24	6,516	31.9	104,691	10.2	
25–29	5,997	29.4	288,020	27.9	
30–34	3,880	19.0	390,930	37.9	
35–39	1,456	7.1	195,834	19.0	
≥40	232	1.1	36,026	3.5	
Pre-pregnancy Body Mass Index (kg/m^2^)^b^					
Underweight (<18.5)	1,733	10.1	52,055	5.8	<0.0001
Normal weight (18.5–24.9)	8,662	50.6	484,620	53.9	
Overweight (25–29.9)	3,584	20.9	208,563	23.2	
Obese (≥30)	3,148	18.4	154,274	17.2	
Missing	3,283	16.1	131,848	12.8	
Rural residence	4,103	20.1	113,580	11.0	<0.0001
Neighborhood income					
Lowest quintile	6,987	34.2	187,459	18.2	<0.0001
Medium-low quintile	4,607	22.6	193,627	18.8	
Middle quintile	3,962	19.4	222,405	21.6	
Medium-high quintile	3,258	16.0	246,890	23.9	
Highest quintile	1,596	7.8	180,979	17.6	
Nulliparous	11,518	56.4	440,622	42.7	<0.0001
Infant sex					
Female	9,939	48.7	503,829	48.9	0.9
Male	10,469	51.3	527,446	51.1	
Prenatal tobacco use	11,232	55.0	80,379	7.8	<0.0001
Prenatal alcohol use	2,768	13.6	17,417	1.7	<0.0001
Other substance use^c^	2,667	13.1	9,574	0.9	<0.0001
Pre-existing diabetes	118	0.6	5,834	0.6	0.8
Gestational diabetes	986	4.8	66,991	6.5	<0.0001
Pre-existing hypertension	82	0.4	4,750	0.5	0.2
Gestational hypertension	616	3.0	30,375	3.0	0.5
Pre-eclampsia/Eclampsia	445	2.2	20,153	2.0	0.02

Note: Includes pregnant individuals who delivered both live and stillbirths.

^a^ Frequencies shown for cannabis use by each fiscal year.

^b^ Frequencies shown exclude missing values due to 13% missing in the total sample.

^c^ Includes solvents, cocaine, methadone, heroin and opioids.

Table 2 summarizes the prevalence and odd ratios for each infant and maternal outcome by prenatal cannabis use. Pregnant individuals who used cannabis had higher rates of LBW, PTB and SGA births. Adjusted regression models indicate that pregnant individuals who used cannabis had 85% higher odds of PTB, 73% higher odds of VPTB and 90% higher odds delivering a LBW infant. There was also 21% higher odds of an infant being SGA and 71% higher odds of any major congenital anomaly. No association was found for stillbirth. For maternal outcomes, prenatal cannabis use was associated with increased risk of gestational diabetes and caesarean section but there was no statistically significant association with gestational hypertension. Only SGA and SPTB had statistically significant variation by infant sex, however, there was overlap in confidence intervals between infant sex strata. As shown in S2 Table, sub-analyses of pregnant individuals who only used cannabis did not eliminate associations. With the exception of LGA, we also didn’t find a difference in odds ratios between those who used all substances versus cannabis alone. We found that the magnitude of confounding needed to explain our observed associations ranged between 1.5 for SGA to 3.5 for preterm birth (S3 Table).

**Table 2:** Associations between cannabis use in pregnancy and maternal and infant outcomes, BC, ON and NL, 2012–2019.

Maternal and Infant Outcomes	Cannabis use		No cannabis use		p-value	Unadjusted Odds Ratios (95% CI)	Adjusted Odds^a^ Ratios (95% CI)	Adjusted Odds^a^ Ratios (95% CI)–Female Infant	Adjusted Odds^a^ Ratios (95% CI)–Male Infant	Interaction p-value
	n	%	n	%						
Preterm (<37 weeks)	1751	9.1	41128	4.2	<0.0001	2.30	1.85	1.88	1.82	0.05
						[2.19–2.42]	[1.74–1.94]	[1.74–2.04]	[1.69–1.96]	
Spontaneous	1097	5.7	24004	2.4	<0.0001	2.47	1.80	1.94	1.70	0.04
						[2.32–2.63]	[1.68–1.93]	[1.76–2.14]	[1.55–1.86]	
Medically	654	3.4	17124	1.7	<0.0001	2.06	1.94	1.80	2.06	0.1
Indicated						[1.90–2.23]	[1.77–2.12]	[1.59–2.05]	[1.83–2.30]	
Very preterm (<32 weeks)	198	1.0	3992	0.4	<0.0001	2.56	1.73	1.87	1.62	0.3
						[2.21–2.95]	[1.48–2.02]	[1.50–2.33]	[1.31–2.00]	
Low birthweight (<2500g)	1292	6.7	23942	2.4	<0.0001	2.89	1.90	1.88	1.93	0.7
						[2.73–3.06]	[1.79–2.03]	[1.73–2.05]	[1.77–2.11]	
SGA (<10^th^ percentile)	2360	12.3	73128	7.4	<0.0001	1.72	1.21	1.28	1.16	0.03
						[1.65–1.80]	[1.16–1.27]	[1.20–1.37]	[1.08–1.23]	
LGA (>90^th^ percentile)	1547	8.1	95430	9.7	<0.0001	0.86	1.06	1.09	1.03	0.3
						[0.82–0.91]	[1.01–1.12]	[1.01–1.18]	[0.96–1.12]	
Stillbirth	60	0.3	1997	0.2	0.001	1.53	1.15	1.14	1.13	1.0
						[1.18–1.98]	[0.86–1.53]	[0.74–1.75]	[0.77–1.66]	
Any major congenital anomaly	246	1.2	6287	0.6	<0.0001	1.99	1.71	1.89	1.63	0.3
						[1.75–2.26]	[1.49–1.97]	[1.51–2.36]	[1.38–1.93]	
Caesarean section	4324	22.9	240013	24.7	<0.0001	0.90	1.13	*	*	*
						[0.87–0.93]	[1.09–1.17]			
Gestational diabetes	883	4.6	56317	5.7	<0.0001	0.79	1.32	*	*	*
						[0.74–0.85]	[1.23–1.42]^b^			
Gestational hypertension	455	2.4	21231	2.2	0.048	1.10	1.10	*	*	*
						[1.001–1.21]	[0.99–1.22]^c^			

*Comparisons by infant sex were only conducted for infant outcomes.

^a^ Models adjusted for other substance use, maternal age, pre-pregnancy BMI, rural maternal residence, parity, QAIPPE score, pre-existing diabetes, chronic hypertension, gestational diabetes and gestational hypertension.

^b^ Models adjusted for other substance use, maternal age, pre-pregnancy BMI, rural maternal residence, parity, QAIPPE score, pre-existing diabetes, chronic hypertension and gestational hypertension.

^c^ Models adjusted for other substance use, maternal age, pre-pregnancy BMI, rural maternal residence, parity, QAIPPE score, pre-existing diabetes, chronic hypertension and gestational diabetes.

## Discussion

This study is the first to combine population-based data from Canadian jurisdictions to investigate the effect of prenatal cannabis use on maternal and infant outcomes. The prevalence of cannabis use in our cohort was approximately 2% which is similar to what has been reported previously by Ontario and British Columbia during the same period (1.3% and 2.4%, respectively) [19, 20]. Yearly trends of perinatal outcomes for the provinces included in our cohort have been previously published elsewhere [21–23]. Pregnant individuals who used cannabis were younger and less likely to have a normal pre-pregnancy BMI, and had higher rates of concurrent substance use, particularly tobacco. After adjusting for other substance use and confounders, we found positive associations between prenatal cannabis use and LBW, PTB, SPTB, MPTB, VPTB, SGA, major congenital anomalies, caesarean section and gestational diabetes. Associations remained in a sub-sample of pregnant individuals who reported only using cannabis. Unmeasured confounders would need to be associated with both cannabis use and outcomes at a magnitude of greater than 2 for most outcomes to fully explain the observed results.

Our results are consistent with previous research showing an association between prenatal cannabis use and LBW, PTB and SGA births [19, 24]. Our analysis of data from multiple provinces also builds on previous work using BORN Ontario perinatal data (up to 2017) which had similar findings in relation to PTB and SGA [20]. Previous research has shown that cannabis use increases plasma cortisol levels in pregnant individuals with higher levels of cortisol increasing the risk for PTB [6, 25–27]. We found some evidence that the effects of prenatal cannabis use on spontaneous PTB and SGA were more pronounced in female infants. These results did not corroborate with previous research showing that in-utero cannabis exposure adversely affects male offspring but not female offspring with regards to LBW and is not associated with length of gestation [12, 13]. However, previous research applied additive instead of multiplicative interactive effects which may account for the differences observed.

Our results indicate an association between major congenital anomalies and prenatal cannabis use. Currently, the evidence is mixed and mostly limited to ecological studies. A more recent study, also using data from BORN Ontario for 2012 to 2018 found positive associations between maternal cannabis use and gastroschisis [28]. Additionally, there is evidence for the development of ventricular septal heart defects, esophageal atresia and diaphragmatic hernia [29–31].

Differences in data collection practices between jurisdictions posed a challenge for combining data sources and limited the number of jurisdictions that could participate in the study. We were not able to include late terminations, thereby limiting our analysis to congenital anomalies among live and stillbirths, and resulting in incomplete stillbirth records. It is possible that this may underestimate the magnitude of effect for congenital anomalies and stillbirth outcomes. Self-reported substance use may be underreported due to social desirability bias and providers are not required to ask about use. Women who use substances may be hesitant to attend prenatal care visits out of fear that their children will be removed from their care. We were not able to account for possible differences in prenatal care utilization in our analysis. Additionally, records with unknown cannabis use are reported as “no use” in some jurisdictions leading to potential misclassification. However, this would mean that the true magnitude of effect is actually greater than what is observed since it is unlikely that women who don’t use cannabis would report that they do. Therefore any misclassification would most likely result in exposed women being included in the reference group, thereby underestimating the magnitude of effect towards the null. This limitation is present for all substance use data collected as women, even if asked universally, may not be willing to disclose their use to a provider. Also, we do not have data on the dose, duration, frequency or route of exposure. However, due to the variability in the concentration of tetrahydrocannabinol in cannabis products and different routes of exposure, correct measures of dosage for cannabis use based on route of exposure have not yet been established and require additional development before implementation is possible. Due to ethical concerns, it is not recommended to universally screen pregnant women for substance use using urine toxicology testing in the context of routine care. Examining the association between any cannabis use and adverse outcomes is the most conservative approach as any observed association can be assumed to be underestimated due to the inclusion of varying degrees of cannabis dose, frequency, route of exposure and duration of use during pregnancy reported by the exposed group. Furthermore, the reason for use is not documented limiting the ability to account for other confounders including depression which pregnant individuals have reported using cannabis to treat [32] and which is also associated with caesarean section, PTB and LBW [33, 34].

Our study includes a large population-based cohort which limits selection bias. While we were not able to capture data from all jurisdictions across Canada, our cohort includes approximately half of all Canadian births as ON represents 40% of births in Canada while BC and NL account for approximately 13% of births.

Research and surveillance of prenatal cannabis use and associated outcomes would be strengthened by standardizing data collection and reporting practices across Canadian jurisdictions. Key strategies to support data collection include addressing the barriers and facilitators to implementing routine screening practices including providers’ lack of knowledge regarding the evidence associated with prenatal cannabis use, perceptions that cannabis is less harmful than other substances, and a focus on the legal consequences of use when counselling patients [35]. Further research is needed beyond the immediate newborn period, as researchers have identified concerning associations between cannabis use during pregnancy and adverse childhood outcomes including autism spectrum disorder [36]. Greater efforts are required to inform the public, to communicate evidence-based information during prenatal visits and to support shared decision making. Health care providers need to make a concerted effort to create a safe space to engage in respectful, trauma-informed discussion especially given that experiences of violence and abuse are more common among pregnant individuals who use substances [37]. Providers can refer to existing discussion guides to counsel patients [38].

Prenatal cannabis use is associated with PTB, LBW, SGA and major congenital anomalies in the newborn. Additional measures are needed to inform the public and providers of the inherent risks of cannabis exposure in pregnancy. Enhancing capacity to collect national data is an important priority while simultaneously promoting trauma-informed approaches to screening and intervention for this population.

## Supporting information

S1 Table Summary of ICD-10-CA codes used to define pregnancy characteristics. (DOCX)

S2 Table Associations of prenatal cannabis use and maternal and newborn outcomes among pregnant individuals who only used cannabis compared to all substance use. (DOCX)

S3 Table Estimation of the effect of unmeasured confounding on the observed associations between cannabis use and maternal and newborn outcomes. (DOCX)
