# Supplementary material for: Cannabis use in pregnancy and maternal and infant outcomes: A Canadian cross-jurisdictional population-based cohort study
Source: PLoS One. 2022 Nov 23;17(11):e0276824. doi: 10.1371/journal.pone.0276824 (PMC9683571; doi:10.1371/journal.pone.0276824)
Supplement: S1 Table — (DOCX) [file pone.0276824.s001.docx]

Supplemental Table 1: Summary of ICD-10-CA codes used to define pregnancy characteristics

| Pregnancy Characteristics | ICD-10-CA Codes |
| --- | --- |
| Pre-existing diabetes | O24.501, O24.502, O24.503, O24.504, O24.509, O24.601, O24.602, O24.603, O24.604, O24.609, O24.701, O24.702, O24.703, O24.704, O24.709 |
| Gestational diabetes | O24.801, O24.802, O24.803, O24.804, O24.809 |
| Pre-existing hypertension | O10.001, O10.002, O10.003, O10.004, O10.009, O10.101, O10.102, O10.103, O10.104, O10.109, O10.201, O10.202, O10.203, O10.204, O10.209, O10.301, O10.302, O10.303, O10.304, O10.309, O10.401, O10.402, O10.403, O10.404, O10.409, O10.901, O10.902, O10.903, O10.904, O10.909 |
| Gestational hypertension | O13.001, O13.002, O13.003, O13.004, O13.009 |
| Pre-eclampsia | O14.001, O14.002, O14.003, O14.004, O14.009, O14.101, O14.102, O14.103, O14.104, O14.109, O14.201, O14.202, O14.203, O14.204, O14.209, O14.901, O14.902, O14.903, O14.904, O14.909, O11.001, O11.002, O11.003, O11.004, O11.009 |
| Eclampsia | O15.001, O15.101, O15.202, O15.003, O15.103, O15.204, O15.909 |
| Major congenital anomalies | Q00, Q05 (if not Q00.0), Q01, Q03, Q02, Q04.1, Q04.2, Q11.0, Q11.1, Q11.2, Q16.0, Q17.2, Q30.0, Q20.0, Q20.1, Q20.3, Q20.5, Q21.2, Q21.3, Q23.4, Q25.1, Q35(excluding Q35.7), Q36, Q37, Q39.0-Q39.4, Q41, Q42.0-Q42.3, Q43.1, Q44.2, Q60.0-Q60.2, Q61.1-Q61.5, Q61.8, Q61.9, Q64.1, Q64.2, Q64.3, Q53.1, Q53.2, Q53.9, Q54 (excluding Q54.4), Q64.0, Q56, Q71-Q73, Q79.0, Q79.2, Q79.3, Q90, Q91.4-Q91.7, Q91.0-Q91.3, Q96 |
