# Supplementary material for: Cannabis use in pregnancy and maternal and infant outcomes: A Canadian cross-jurisdictional population-based cohort study
Source: PLoS One. 2022 Nov 23;17(11):e0276824. doi: 10.1371/journal.pone.0276824 (PMC9683571; doi:10.1371/journal.pone.0276824)
Supplement: S2 Table — (DOCX) [file pone.0276824.s002.docx]

Supplemental Table 2: Associations of prenatal cannabis use and maternal and newborn outcomes among pregnant individuals who only used cannabis compared to all substance use

| Maternal and Infant Outcomes | Cannabis use only  Adjusted Odds Ratios  (95% CI) | All substance use  Adjusted Odds Ratios  (95% CI) |
| --- | --- | --- |
| Preterm (<37 weeks) | 2.02 [1.86-2.21] | 1.85 [1.74-1.94] |
| Spontaneous | 2.02 [1.81-2.25] | 1.80 [1.68-1.93] |
| Medically Indicated | 2.05 [1.79-2.34] | 1.94 [1.77-2.12] |
| Very preterm (<32 weeks) | 2.04 [1.59-2.60] | 1.73 [1.48-2.02] |
| Low birthweight (<2500g) | 1.88 [1.68-2.11] | 1.90 [1.79-2.03] |
| SGA (<10^th^ percentile) | 1.12 [1.03-1.22] | 1.21 [1.16-1.27] |
| LGA (>90^th^ percentile) | 1.23[1.14-1.32] | 1.06 [1.01-1.12] |
| Stillbirth | 1.39 [0.89-2.18] | 1.15 [0.86-1.53] |
| Any major congenital anomaly | 1.66 [1.33-2.07] | 1.71 [1.49-1.97] |
| Caesarean delivery | 1.22 [1.15-1.29] | 1.13 [1.09-1.17] |
| Gestational diabetes | 1.33 [1.20-1.46] | 1.32 [1.23-1.42] |
| Gestational hypertension | 1.23 [1.07-1.41] | 1.10 [0.99-1.22] |
