# Supplementary material for: Cannabis use in pregnancy and maternal and infant outcomes: A Canadian cross-jurisdictional population-based cohort study
Source: PLoS One. 2022 Nov 23;17(11):e0276824. doi: 10.1371/journal.pone.0276824 (PMC9683571; doi:10.1371/journal.pone.0276824)
Supplement: S3 Table — (DOCX) [file pone.0276824.s003.docx]

Supplemental Table 3: Estimation of the effect of unmeasured confounding on the observed associations between cannabis use and maternal and newborn outcomes

| Maternal and Infant Outcomes | Cannabis use only  E-value | All substance use  E-value |
| --- | --- | --- |
| Preterm (<37 weeks) | 3.5(3.5) | 3.1(2.9) |
| Spontaneous | 3.5(3.0) | 3.0(2.7) |
| Medically Indicated | 3.5(3.0) | 3.3(2.9) |
| Very preterm (<32 weeks) | 3.5(2.5) | 2.9(2.3) |
| Low birthweight (<2500g) | 3.2(2.7) | 3.2(3.0) |
| SGA (<10^th^ percentile) | 1.5(1.2) | 1.7(1.6) |
| LGA (>90^th^ percentile) | 1.8(1.5) | 1.3(1.1) |
| Stillbirth | 2.1(1.0) | 1.6(1.0) |
| Any major congenital anomaly | 2.7(2.0) | 2.8(2.3) |
| Caesarean delivery | 1.7(1.6) | 1.5(1.4) |
| Gestational diabetes | 2.0(1.7) | 2.0(1.8) |
| Gestational hypertension | 1.8(1.3) | 1.4(1.0) |
